# Eutrophication has no short-term effect on the *Cymbastela stipitata* holobiont

**DOI:** 10.3389/fmicb.2014.00216

**Published:** 2014-05-14

**Authors:** Heidi M. Luter, Karen Gibb, Nicole S. Webster

**Affiliations:** ^1^North Australia Marine Research Alliance and Research Institute for the Environment and Livelihoods, Charles Darwin UniversityDarwin, NT, Australia; ^2^Research Institute for the Environment and Livelihoods, Charles Darwin UniversityDarwin, NT, Australia; ^3^Australian Institute of Marine ScienceTownsville, QLD, Australia

**Keywords:** *C. stipitata*, sponge, microbial community, sponge-microbe symbiosis, sewage effluent

## Abstract

Levels of nitrogen in coastal areas have been rapidly increasing due to accumulative inputs of sewage and terrigenous sediments carrying fertilizers. Sponges have an immense filtering capacity and may be directly impacted (positively or negatively) by elevated concentrations of nitrogen. Sponges also host a wide diversity of microbes involved in nitrogen metabolism, yet little is known about the effects of nitrogen loading on these symbiotic partnerships. Manipulative experiments were undertaken to examine the potential effects of excess nitrogen (up to 240 μM) on microbial symbiosis in the abundant sponge species *Cymbastela stipitata.* Microbial composition and activity were examined using 454-pyrotag sequencing of DNA- and RNA-derived samples. Despite the high levels of nitrogen exposure (up to 124-fold above ambient), sponges appeared visibly unaffected at all treatment concentrations. At the phylum level, the microbial community was consistent between all sponge samples regardless of nitrogen treatment, with *Cyanobacteria* and *Thaumarchaeota* being the dominant taxa. Higher microbial diversity was observed at the operational taxonomic units (OTU) level (97% sequence similarity), with only 40% of OTUs shared between samples from all treatments. However, a single cyanobacterial OTU dominated the community of all individuals (average 73.5%) and this OTU did not vary with nitrogen treatment. The conserved microbial community in all sponges irrespective of nitrogen treatment highlights the stability of the sponge-microbe relationship and indicates that the holobiont is resistant to short pulses of nitrogen at levels mimicking sewage effluent.

## INTRODUCTION

Nitrogen is generally considered limiting in marine systems although levels in coastal areas have been increasing due to inputs of terrigenous sediments, carrying fertilizers and sewage (Brodie and Mitchell, 2005; Burford et al., 2008). Increased eutrophication has led to the degradation of coastal reefs worldwide via: (1) increased nutrient levels causing trophic shifts, (2) light attenuation from increased turbidity which reduces photosynthesis, and (3) increased sedimentation which reduces larval settlement and increases mortality (Fabricius, 2011). In particular, sewage effluent has been linked to phytoplankton blooms (Caperon et al., 1971) and increased benthic microalgae biomass (Smith et al., 1981) both of which contribute to reef degradation. Darwin Harbor, located in the Northern Territory of Australia, has generally been considered a pristine Harbor with low nutrient inputs (McKinnon et al., 2006). However, recent studies have linked high nitrogen levels in tidal creeks to sewage effluent (Smith et al., 2011; Burford et al., 2012), making point-source sewage discharges and increased urbanization the largest water quality management issues in the Harbor (Fortune and Maly, 2009).

Sponges in Darwin Harbor comprise a diverse and ecologically significant component of benthic communities (Alvarez and Hooper, 2011). Due to their immense filtering capacity [an individual sponge can filter 1000s of liters of seawater per day (Reiswig, 1971)], sponges will be highly exposed to these elevated levels of nitrogenous compounds. Other benthic invertebrates, such as corals, display adverse effects from increased nutrients including dissolved inorganic nitrogen levels above 4 μM which has been shown to cause reduced reproduction and growth (Koop et al., 2001) and increases in disease severity and progression (Bruno et al., 2003; Voss and Richardson, 2006). However, it is still unclear whether sponges share this sensitivity to elevated levels of nitrogen.

Sponges are amongst the oldest metazoans and their evolutionary success has been linked to their intimate association with microorganisms (Webster and Taylor, 2012). Sponges host a diverse array of microorganisms, with 32 different bacterial phyla and both major lineages of archaea identified to date (Taylor et al., 2007; Schmitt et al., 2012; Webster and Taylor, 2012). While it is known that sponges host microorganisms involved in nitrogen metabolism (Wilkinson and Fay, 1979; Southwell et al., 2008; Weisz et al., 2008; Hoffmann et al., 2009; Schläppy et al., 2009; Mohamed et al., 2010; Off et al., 2010), little is known about the effects of nitrogen loading on the sponge-microbe symbiosis, particularly at the high levels found in sewage effluent. However, in a recent study of sponges in Brazil, a higher diversity of sponge-associated *Crenarchaeota* was observed from the more polluted areas of Rio de Janeiro, suggesting that sponges may have the ability to alter their community in response to eutrophic environments (Turque et al., 2010).

Here we exposed the sponge *Cymbastela stipitata* (Bergquist and Tizard, 1967), one of the most abundant sponge species found in Darwin Harbor (Alvarez and Hooper, 2009), to concentrations of nitrogen ranging from 2 μM (ambient) to 240 μM and performed 454-pyrotag sequencing of the 16S rRNA gene in DNA- and RNA-derived samples to determine how anthropogenic inputs of nitrogen affect sponge microbial symbiosis.

## MATERIALS AND METHODS

### SPONGE COLLECTION

Individuals of *C. stipitata* were collected from Channel Island, Northern Territory (12° 33′ 02. 4′′ S, 130° 52′ 31. 3′′ E) during the lowest tide of June 2013. Sponges were collected on foot using a hammer and chisel, taking special care to ensure some substrate was included to avoid injuring the sponge tissue. Sponges were immediately transported to the Darwin Aquaculture Centre (DAC) on Channel Island where they were placed into 12 × 30 l flow-through aquaria (flow rate of 400 ml min^-1^) and maintained under natural lighting conditions. DAC seawater supply is pumped in from a pipe 40 m off Channel Island and filtered to 5 μm to remove large particulates, yet leaving sponges with a sufficient food supply (Reiswig, 1971). Sponges were kept under these conditions for one week to allow them to acclimatize prior to commencing the experiment.

### EXPERIMENTAL DESIGN

To assess the effect of elevated nitrogen levels on the sponge-microbe symbiosis, *C. stipitata* individuals were exposed to four different nutrient treatments using the water-soluble plant fertilizer Thrive^®^ (Yates, NPK; 27:5:5:9 and trace elements). For the first two days, three different stock concentrations (low, medium and high) of Thrive were continuously pumped into treatment tanks at 4 ml min^-1^ at final total inorganic nitrogen concentrations of 2 μM (Ambient), 120 μM (Low), 160 μM (Medium) and 240 μM (High), mimicking a sewage discharge event (Power and Water Corporation, 2006). After 48 h, Thrive input was decreased for an additional 5 days (2 μM (Ambient), 3, 5, and 15 μM), reflecting a flood plume event (Kroon et al., 2012). The experimental design comprised three replicate tanks per treatment, each holding three sponges. One individual sponge per tank/treatment was sacrificed at each sampling day: 0, 2, and 7. Samples were snap frozen in liquid nitrogen and stored at -80°C for further analysis. Histological specimens were prepared as previously described in Luter et al. (2011) to compare the internal tissue structure between samples in different treatments.

After the first day of the experiment, one of the sponges from the high nitrogen treatment displayed substantial tissue discoloration. It was immediately removed from the experiment to avoid compromising the health of the other sponges in the tank.

### NUTRIENT ANALYSIS

Dissolved nutrient levels [ammonium, nitrite, nitrate, phosphate, dissolved organic carbon (DOC)] were monitored throughout the experiment to ensure treatment levels were maintained. Seawater samples were hand filtered through 0.45 μm cellulose acetate filter cartridges (Sartorius MiniStart) into acid-washed 15 ml tubes and stored at -20° C prior to dissolved nutrient analysis. Filtered samples for DOC were acidified with 100 μl HCl prior to freezing. Nutrient samples were analyzed by the analytical services laboratory at the Australian Institute of Marine Science (AIMS, Townsville).

### DNA/RNA EXTRACTIONS AND 454-PYROSEQUENCING

DNA and RNA were simultaneously extracted from individual sponge tissue samples (≤30 mg) using the AllPrep DNA/RNA Mini kit (Qiagen), following the manufacture’s protocols. DNA contamination was eliminated from RNA samples using the TURBO DNA-free kit (Ambion). The quantity and purity of DNA and RNA was determined using gel electrophoresis [1.1% agarose gels containing GelRed (Biotium)] and a NanoDrop 2000 spectrophotometer (Thermo Scientific). DNA and RNA were sent to MR DNA (http://www.mrdnalab.com) where RNA was reverse transcribed into cDNA and the 16S rRNA gene was amplified using both bacterial (27Fmod and 530 R; Dowd et al., 2008) and archaeal [arch344F (Giovannoni et al., 1988) and arch915R (Stahl and Amann, 1991)] primers and sequenced using the Roche GS-FLX platform. Raw sequence data have been submitted to the NCBI Sequence Read Archive (SRA) under the accession number: SRP038768.

### PROCESSING OF RAW SEQUENCE DATA AND TAXONOMIC ASSIGNMENT

Pyrosequencing flowgram files (SFF) were processed using Mothur (Schloss et al., 2009). Flowgrams were filtered and denoised using the AmpliconNoise (Quince et al., 2011) function in Mothur. If sequences were less than 200 bp, contained ambiguous characters, or had homopolymers longer than 8 bp, more than one MID mismatch, or more than two mismatches to the reverse primer sequence, they were removed from the analysis. Sequences deemed unique by Mothur were aligned against a SILVA alignment (http://www.mothur.org/wiki/Silva_reference_alignment). Chimeric sequences were removed using UCHIME (Edgar et al., 2011), and samples were sub sampled down to the lowest read number and grouped into 97% OTUs based on pairwise distance matrices created in Mothur. OTUs were taxonomically classified at 97% sequence similarity with the RDP Classifier (Wang et al., 2007) using the SILVA database as a reference.

### DATA ANALYSES

The unconstrained principal coordinate analysis (PCO) was used to visually compare communities, while PERMNOVA was used to test differences in community structure. All analyses were performed using PRIMER/PERMANOVA+ (Plymouth, UK).

### CLONE LIBRARY CONSTRUCTION AND PHYLOGENETIC ANALYSIS

A clone library was constructed to obtain a full length sequence of the dominant cyanobacterial OTU identified in 454-pyrosequencing analysis. The 16S rRNA gene was amplified using the universal bacterial primers 63f (Marchesi et al., 1998) and 1492r (Lane, 1991). PCR reactions contained 5 μl Bio-X-Act buffer, 0.10 μl of each primer (100 pmol μl^-^^1^), 0.4 μl BSA (10 mg ml^-1^), 0.5 μl Bio-X-act short DNA polymerase (Bioline, London, UK) and 1 μl DNA. Reactions were made up to 50 μl using high pure water. The conditions consisted of 1 cycle at 95°C for 5 min, followed by 30 cycles at 95°C for 30 s, 56°C for 30 s and 72°C for 2 min, and a final elongation at 72°C for 10 min. PCR products from the three control sponges samples were pooled and gel purified using a Wizard SV Gel and PCR Clean-Up Kit following the manufacture’s protocol (Promega, Madison, WI, USA). The purified PCR product was cloned using a TOPO-TA cloning kit (Invitrogen, Carlsbad, CA, USA) according to the manufacture’s protocol. Plasmids were checked for inserts by PCR amplification using M13 forward and reverse primers. Restriction digests using *HAEIII* and *HhaI* (New England Biolabs Inc., Ipswich, MA, USA) were used to determine OTUs. Twenty clones were screened and triplicates of the dominant OTU pattern was sent for sequencing using the forward and reverse primers at the Australian Genomic Research Facilities (Brisbane, Australia). The sequence was submitted to Genbank under the accession number: KJ719259.

The clone sequence was compared to available databases by using BLAST to determine the nearest relatives and the percent similarity. Sequences from the nearest relatives, other sponge-specific cyanobacterial sequences and sequences representing the diversity of *Cyanobacteria* were compiled and aligned using the SINA web aligner (Pruesse et al., 2012). Maximum likelihood analysis and tree construction were performed using MEGA version 6.06 (Tamura et al., 2013), following the procedures of Hall (2013). The Kimura 2-parameter model with a gamma distribution (four categories) and invariant sites was found to be the most appropriate model. The reliability of the tree was tested by computing 500 bootstrap replicates starting with a neighbor-joining tree, and using the nearest-neighbor interchange (NNI) tree search option.

### CHLOROPHYLL (CHL) A EXTRACTION AND ANALYSIS

As an estimate of potential photosymbiont abundance, Chl *a* was extracted from pre-weighed tissue samples (stored at -80°C) using 90% acetone:water overnight at 4°C. Samples were centrifuged and the supernatant was transferred to a spectrophotometer cuvette. Absorbance readings were taken at 750, 664, 647, and 630 nm using the Helios Gamma2 spectrometer. Chl *a* levels were calculated following equations from Parsons et al. (1984) and standardized using tissue weight.

## RESULTS

**FIGURE 1:**
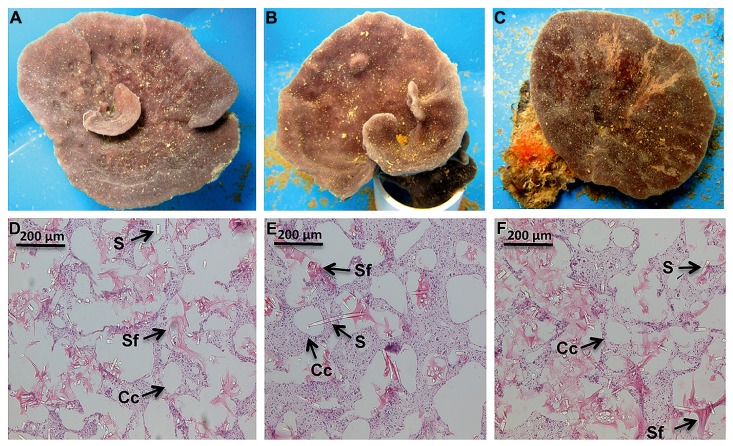
**Representative images, both external and internal, of *C. stipitata* from the day 0 control (A,D), *T* = 2, High (B,E) and *T* = 7, High (C,F)**. A representative choanocyte chamber (Cc), sponge fiber (Sf), and spicule (S) are depicted.

Nutrient levels from the ambient treatment were used to determine fold differences in total inorganic nutrient levels achieved in the treatment exposures over the course of the experiment. During the first 48 h of the experiment, *C. stipitata* individuals in the highest treatment were exposed to more than a 100-fold enrichment of total inorganic nitrogen (**Table 1**). For the remainder of the experiment levels were reduced, with sponges in the highest treatment exposed to a 13.5-fold enrichment of total inorganic nitrogen (**Table 1**. Despite the extremely high nitrogen exposures, *C. stipitata* individuals showed no visible effects on the external (**Figures 1A–C**) or internal (**Figures 1D–F**) tissues.

**Table 1:** Nutrient parameters measured over the course of the 7 day experiment (three replicate water samples averaged per treatment).

Sample	NPOC (mg/l)	NH_4_ (μM)	PO_4_ (μmol/l)	NO_2_ + NO_3_ (μM)	NO_2_ (μM)	Total Inorganic N (μM)
Control- Ambient (*T* = 0–7)	1.1 ± 0.1	0.6 ± 0.1	0.4 ± 0.04	1.4 ± 0.4	0.0 ± 0.01	2.0 ± 0.5
Low (*T* = 1,2)	6.1 ± 3.1	114.8 ± 39.8	68.6 ± 26.8	5.8 ± 1.1	0.2 ± 0.1	120.6 ± 40.9
Medium *(T* = 1,2)	10.9 ± 5.6	150.2 ± 115.7	82.1 ± 39.3	4.9 ± 2.6	0.1 ± 0.1	155.1 ± 118.3
High (*T* = 1,2)	15.4 ± 4.5	240.3 ± 63.8	121.1 ± 21.6	7.9 ± 1.7	0.1 ± 0.01	248.2 ± 65.5
Low (*T* = 3–7)	1.2 ± 0.1	5.3 ± 1.5	4.4 ± 1.2	1.3 ± 0.1	0.1 ± 0.04	6.7 ± 1.6
Medium (*T* = 3–7)	1.6 ± 0.1	9.4 ± 2.4	8.8 ± 2.4	1.0 ± 0.2	0.1 ± 0.02	10.4 ± 2.6
High (*T* = 3–7)	2.2 ± 0.2	25.8 ± 3.5	20.0 ± 2.1	1.1 ± 0.4	0.1 ± 0.1	27.0 ± 3.9
*in situ channel Is.	1.41 ± 0.1	1.1 ± 0.3	0.9 ± 0.5	1.9 ± 0.2	0.1 ± 0.01	3.1 ± 0.5
**Fold change from Ambient**						
**Sample**	**NPOC (mg/l**)	**NH_4_ (μM)**	**PO_4_ (μM)**	**NO_2_ + NO_3_ (μM)**	**NO_2_ (μM)**	**Total inorganic N (μM**)

Low (*T* = 1,2)	5.5	188.6	171.4	4.1	7.3	60.3
Medium (*T* = 1,2)	9.9	246.7	205.2	3.5	4.5	77.6
High (*T* = 1,2)	14	394.8	302.8	5.6	3.2	124.1
Low (*T* = 3–7)	1.1	8.8	11	0.9	2.9	3.3
Medium (*T* = 3–7)	1.5	15.5	21.9	0.7	1.8	5.2
High (*T* = 3–7)	2	42.4	50	0.8	3.1	13.5

After filtering and quality control, 316,494 bacterial sequences and 252,640 archaeal sequences were obtained from 52 samples (26 DNA- and 26 RNA-derived). Bacterial sequences averaged 6,086 (±3,322 1SD) reads per sample, while archaeal sequences averaged 3,770 (±2,912 1SD) reads per sample. We identified 1,471 bacterial OTUs and 141 archaeal OTUs (97% sequence similarity) spanning nine bacterial phyla and two major lineages of the archaea (*Thaumarchaeota* and *Euryarchaeota*).

**FIGURE 2:**
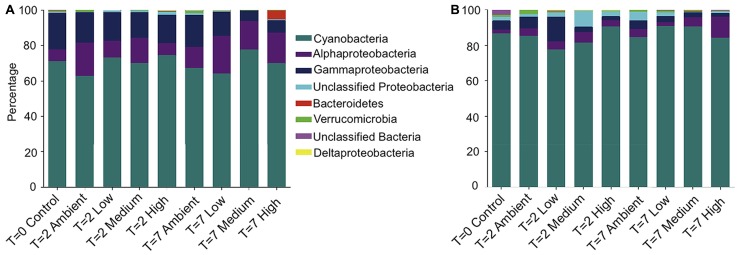
**Relative abundance of each bacterial phyla, plus class for *Proteobacteria*, within each treatment for (A) DNA-derived and (B) RNA-derived sequences**. The three replicate individuals per treatment were averaged. For clarity, only the 50 most abundant operational taxonomic units (OTUs) were used to create the graphs.

Taxonomic assignment of the OTUs revealed that the bacterial community from both the DNA- and RNA-derived samples was conserved between treatments and time points at the phyla level (**Figures 2A,B**). Both communities were dominated by *Cyanobacteria*, with 70% of 16S rRNA-genes, and 86% of transcripts (averaged across treatments) being assigned to *Cyanobacteria*. The next most abundant phyla observed across all DNA- and RNA-derived samples was the *Proteobacteria*, particularly members of the *Alphaproteobacteria* (14 and 4%) and *Gammaproteobacteria* (14 and 5%; **Figures 2A,B**). All other Phyla observed (*Bacteroidetes*, *Deltaproteobacteria*, and *Verrucomicrobia*) occurred in lower abundances and varied between treatments, but not consistently across replicates.

Interestingly, of the 169 cyanobacterial-assigned OTUs, a single OTU (OTU0001) that was 94% similar to the *Cyanobacteria* symbiont of *Mycale (Carmia) hentscheli* (AJ292195) comprised 99% of the total *Cyanobacteria* reads. A similar trend was also observed for the other dominant phyla. OTU0002, which was 98% similar to a Phyllobacterium of *Muricea elongata* (DQ917852) comprised 71% of the *Alphaproteobacteria* reads, while OTU003 comprised 47% of the *Gammaproteobacteria* reads and shared 99% similarity with a symbiont of *C. concentrica* (AY942761).

**FIGURE 3:**
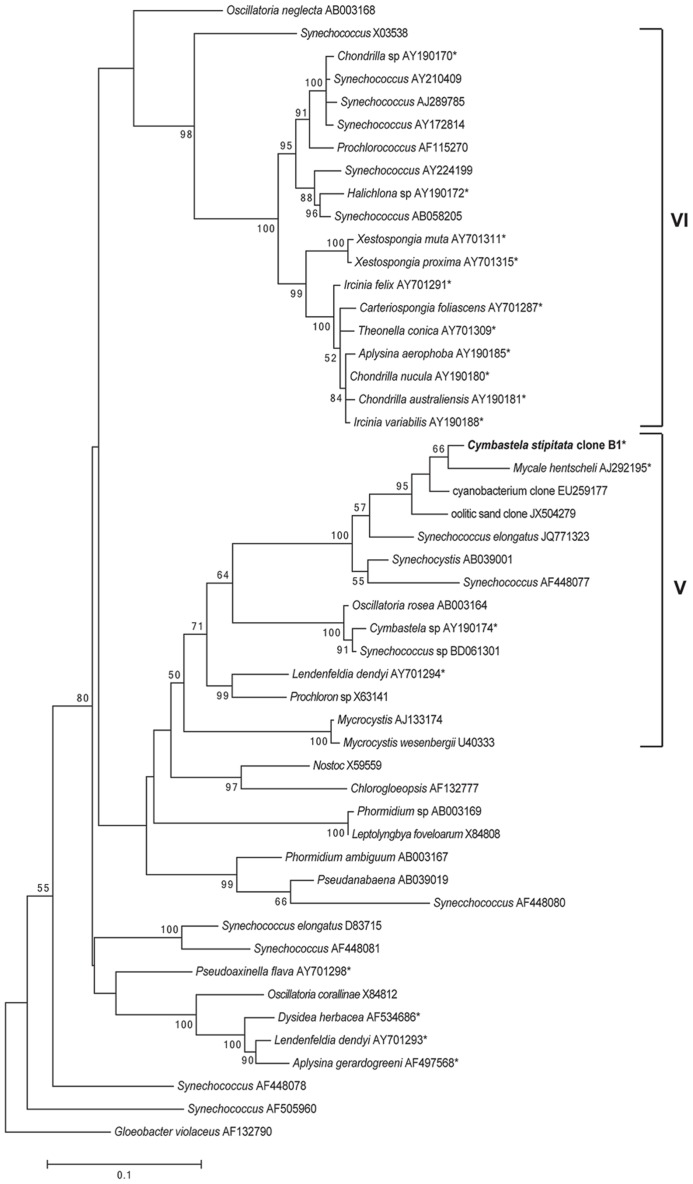
**Maximum-likelihood phylogenetic tree of the *C. stipitata* cyanobacterial clone, indicated by boldfacing, based on 16S rRNA sequences**. Other sponge-associated *Cyanobacteria* are indicated by asterisks (*). The numbers at the nodes are percentages, indicating the levels of bootstrap support based on analysis of 500 resampled data sets. Only values >50% are shown. The scale bar represents 0.1 substitutions per nucleotide position.

Phylogenetic analysis of the full length sequence of the dominant cyanobacterial OTU (OTU0001) revealed its position within group 5 *Cyanobacteria* (**Figure 3**), comprising the *Chroococcales, Oscillatoriales, Pleurocapsales,* and *Prochlorales* (Honda et al., 1999). While most sponge-derived cyanobacteria fall within clusters of sponge-specific sequences (Simister et al., 2012a) and many affiliate with group 6 *Cyanobacteria* (Steindler et al., 2005), symbionts within group 5 *Cyanobacteria* have previously been reported from *Cymbastela* sp., *M. hentsheli*, and *Lendenfeldia dendyi* (**Figure 3**).

**FIGURE 4:**
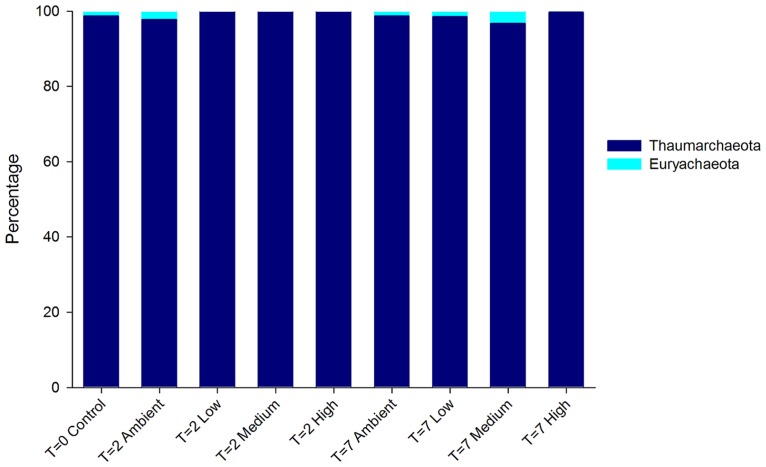
**Relative abundance of archaeal OTUs from DNA-derived sequences**. The three replicate individuals per treatment were averaged.

The DNA-derived archaeal community of *C. stipitata* from every treatment was almost exclusively comprised of *Thaumarchaeota* (**Figure 4**). The other archaeal OTUs represented members of the *Euryachaeota*; however, combined they comprised <1% of the total community. Interestingly, OTUs classified as *Euryachaeota* were not observed in samples from the highest nitrogen treatment. The dominant *Thaumarchaeota* OTU shared 99% similarity with a *Montipora hispida* symbiont (JX021862).

**FIGURE 5:**
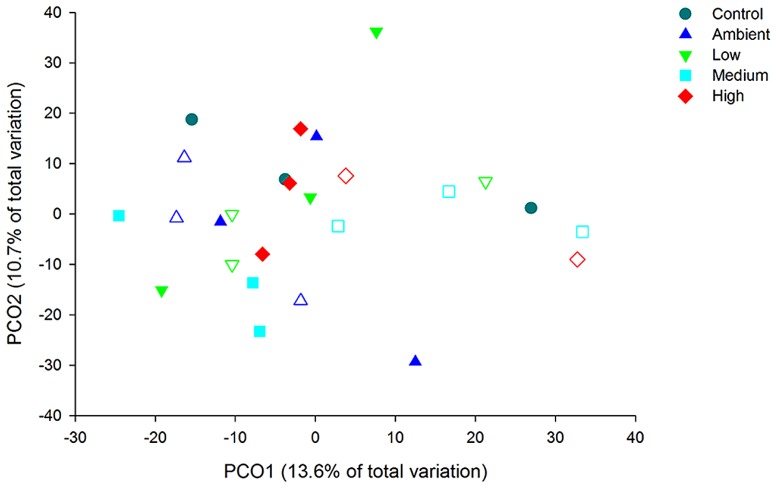
**Principal coordinate analysis (PCO) of the OTUs from the DNA-derived samples**. Control samples are from day 0, solid symbols represent day 2 samples and open symbols represent day 7 samples.

**FIGURE 6:**
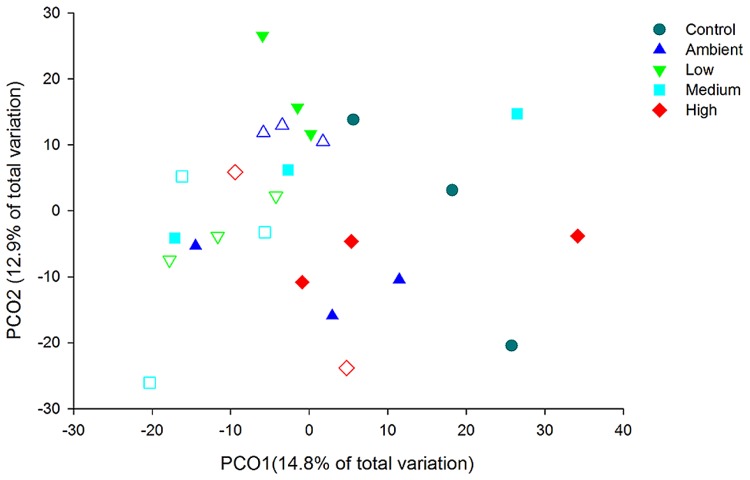
**PCO of the OTUs from the RNA-derived samples**. Control samples are from day 0, solid symbols represent day 2 samples and open symbols represent day 7 samples.

Principal coordinate analysis of the OTUs from DNA-derived samples revealed a large degree of variation between samples, with 27.7% of the total variation in community composition explained within the first two factors (**Figure 5**). All samples shared 40% similarity in community composition and no particular groupings according to nitrogen treatment were evident. The DNA-derived community was not significantly different between nutrient treatments (PERMNOVA, *p* = 0.075). PCO of the OTUs from RNA-derived samples also showed no distinctive clustering of samples according to treatment or time point (**Figure 6**) and explained a lower amount of the total variation in the first two factors (24.3%). The RNA-derived community shared only 20% similarity between all samples and was also not significantly different between nitrogen treatments (PERMNOVA, *p* = 0.216).

**FIGURE 7:**
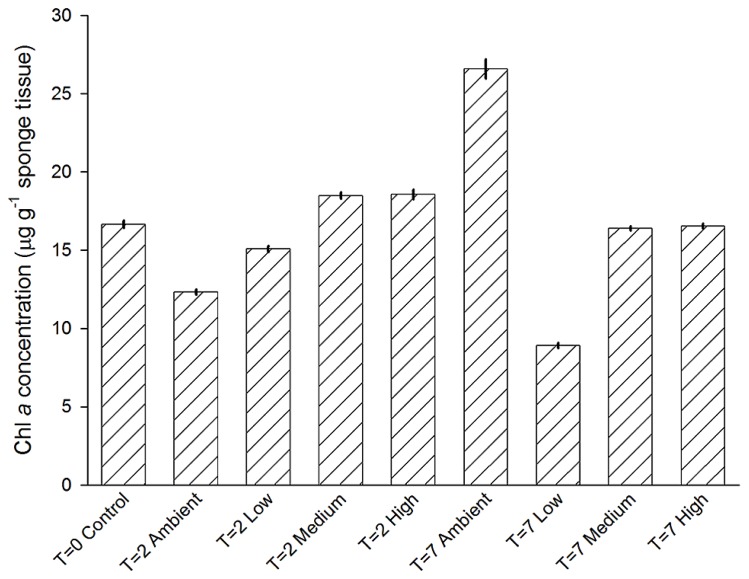
**Mean (±SE) concentration of Chl *a* in *C. stipitata* individuals from each treatment (*n* = 3 for each)**.

Chlorophyll *a* concentrations of individuals among treatments were not significantly different (PERMNOVA, *p* = 0.286). Over the duration of the experiment concentrations ranged from 9 ± 0.2 to 27 ± 0.6 μg Chl *a* g^-1^ sponge tissue (**Figure 7**). Both the lowest and highest levels of Chl *a* were observed at day 7, with sponges from the Low treatment displaying the lowest concentrations, while sponges from the Ambient treatment displayed the highest concentrations.

## DISCUSSION

This is the first study to comprehensively describe the microbial community of *C. stipitata* at the gene and transcript level using 454-pyrotag sequencing. Both the DNA- and RNA-derived samples revealed that the microbial communities (both total and active) were conserved in all individuals at the phyla level and dominated by *Cyanobacteria* and *Thaumarchaeota*, although *Alphaproteobacteria* and *Gammaproteobacteria* were also abundant. Previous studies of sponges from the *Cymbastela* genus described similar microbial compositions, with representatives of *Cyanobacteria, Alphaproteobacteria*, and *Gammaprotobacteria* being reported in both *C. coralliophila* (Webster et al., 2013) and *C. concentrica* (Taylor et al., 2004, 2005). While the representation of phyla was similar across the genus, *Cyanobacteria* appeared to dominate a larger percentage of the total community of *C. stipitata* (70% total, 86% active) than *C. coralliophila* (20%) or *C. concentria* (<5%; Fan et al., 2012), although differences in methodology preclude a direct comparison. While it is clear that the *Cymbastela* genus forms an association with cyanobaterial symbionts, phylogenetic analysis revealed the dominant symbiont of *C. stipitata* to be more closely related to *M. hentscheli* than the other *Cymbastela* species; however, both cyanobacterial symbionts fall into group 5 *Cyanobacteria* (Honda et al., 1999). The archaeal community of the *Cymbastela* genus appears more variable, with metagenomic studies revealing the conditional association of *Thaumarchaeota* in *C. concentrica*, yet, *Thaumarchaeota* were absent in *C. coralliophilia* (Fan et al., 2012). Interestingly, *Cyanobacteria* and *Thaumarchaeota* are well known for their roles in nitrogen metabolism including nitrogen fixation (Wilkinson and Fay, 1979) and ammonia oxidation (Pester et al., 2011), respectively. The dominance of these two groups in the active community of *C. stipitata* suggests they may be playing an important functional role in this sponge.

Examination of the DNA- and RNA-derived microbial communities at the OTU level revealed a low degree of similarity between individual samples. However, given that the majority (73.5%) of the total community (averaged across DNA- and RNA-derived samples) was comprised of a single cyanobacterial OTU (OTU0001), the variation between individuals is likely due to rare OTUs found in lower abundances. The dominance of a single symbiont is not uncommon among sponge species. For instance, a single gammaproteobacterial OTU made up 49% of all 454 tag sequences (Webster et al., 2010) and >90% of all clone library sequences (Luter et al., 2010) in the sponge *Ianthella basta*. Similarly, a specialist *Gammaproteobacteria* symbiont dominated more than 50% of the *Haliclona tubifera* microbial community (Erwin et al., 2011). The presence of a few dominant symbionts in the sponge hosts studied may be a key factor in their ability to withstand a range of environmental stressors. For instance, the three dominant symbionts of *I. basta* remain stable regardless of their disease status (Luter et al., 2010) or exposure to elevated temperature and sedimentation (Luter et al., 2012).

*Cymbastela stipitata* exposed to levels of nitrogen consistent with sewage effluent discharge and flood plume events remained visibly unaffected after 7 days. In addition, phyla level comparisons revealed that both the total and active microbial community was conserved between individuals, regardless of the nitrogen treatment. It is possible that this high tolerance is linked to adaptation mechanisms of the host and/or microbial symbionts that could not be detected with amplicon sequencing. The microbial stability observed in *C. stipitata* during elevated nutrient exposure is consistent with findings for *Rhopaloeides odorabile* (Simister et al., 2012b) and *Aplysina cauliformis* (Gochfeld et al., 2012). However, in both of those studies sponges were exposed to inorganic nitrogen levels 11.4-fold (Simister et al., 2012b) and 4.8-fold (nitrate; Gochfeld et al., 2012) above ambient, whereas sponges from the highest treatment in this experiment experienced enrichments ten times higher (124-fold above ambient), demonstrating an incredibly high resistance to elevated nitrogen concentrations. Given sponges can be abundant on inshore reefs (Wilkinson and Cheshire, 1989) more commonly associated with eutrophic conditions (Brodie and Mitchell, 2005), it is possible that they and their symbionts have developed a tolerance to higher levels of nitrogenous inputs.

This study provides a comprehensive microbial community analysis of an abundant sponge species, revealing the dominance of two symbionts known to be involved in nitrogen metabolism and highlighting the nitrogen tolerance of the *C. stipitata* holobiont.

## Conflict of Interest Statement

The authors declare that the research was conducted in the absence of any commercial or financial relationships that could be construed as a potential conflict of interest.
